# Cytokine pattern during asymptomatic *Anaplasma* spp. infections and effect of co-infections by malaria and helminths in schoolchildren of Franceville, southeastern Gabon

**DOI:** 10.1186/s13071-025-06714-1

**Published:** 2025-03-27

**Authors:** Chérone Nancy Mbani Mpega Ntigui, Sandrine Lydie Oyegue-Liabagui, Jenny Mouloungui-Mavoungou, Nal Kennedy Ndjangangoye, Desly Luide Madoungou Idoumi, Lady Charlene Kouna, Roland Fabrice Kassa Kassa, Nancy Diamella Moukodoum, Steede Seinnat Ontoua, Roméo Karl Imboumy Limoukou, Jean-Claude Biteghe Bi Essone, Alain Prince Okouga, Félicien Bagueboussa, Jean-Bernard Lekana-Douki

**Affiliations:** 1 Unité d’Evolution Epidémiologie et Résistances Parasitaires (UNEEREP), Centre Interdisciplinaire de Recherches Médicales de Franceville (CIRMF), BP 769 Franceville, Gabon; 2 Unité de Recherches d’Analyses Médicales (URAM), Centre Interdisciplinaire de Recherches Médicales de Franceville (CIRMF), BP 769 Franceville, Gabon; 3 Ecole Doctorale Régionale d’Afrique Centrale en Infectiologie Tropicale (ECODRAC), Université des Sciences et Techniques de Masuku, BP 876 Franceville, Gabon; 4 Département de Biologie, Université des Sciences et Techniques de Masuku (USTM), BP 914 Franceville, Gabon; 5 Département de Parasitologie-Mycologie, Université des Sciences de la Santé (USS), BP 4009 Libreville, Gabon

**Keywords:** Asymptomatic, Febrile, *Anaplasma spp* infection, Co-infection with *Plasmodium* and helminths, Cytokines

## Abstract

**Background:**

Asymptomatic infections by *Anaplasma* spp. and the basis of the immune response during these infections have not yet been established. This study investigated the inflammatory cytokine responses during *Anaplasma* spp. infection in school children and the effect of co-infection with *Plasmodium spp*. and helminths.

**Methods:**

Blood and stool samples were taken from children aged 5 to 17 years. Parasitological diagnosis was carried out by RDT and microscopy, while microscopy and PCR were used to diagnose infection by *Anaplasma* spp. Plasma was used for cytokine assays using the ELISA technique.

**Results:**

A total of 219 children were included in the present study, of whom 205 were infected with *Anaplasma* spp. and 14 were uninfected. Levels of IL-6, IL-22 and TGF-β were lower not only in children mono-infected with *Anaplasma spp*. but also in those co-infected with *Anaplasma* spp. and *Plasmodium* spp., *Anaplasma* spp. and helminths, and *Anaplasma* spp., *Plasmodium* spp. and helminths compared to controls. However, higher levels of IL-6 and IL-22 were observed in children mono-infected with *Anaplasma* spp. compared to those co-infected with *Anaplasma* spp. and helminths. The latter group also had lower levels of IL-6, IL-22, TGF-β and IL-10 than children co-infected with *Anaplasma* spp. and *Plasmodium* spp. In addition, children co-infected with *Anaplasma* spp. and helminths had also lower TGF-β and IL-10 levels than children co-infected with *Anaplasma* spp., *Plasmodium* spp. and helminths. An increase of IFN-γ and IL-10 were observed in children co-infected with *Anaplasma* spp. and *Plasmodium* spp. compared to those mono-infected with *Anaplasma* spp. Finally, the results showed that febrile children infected with *Anaplasma* spp. had higher levels of IFN-γ and lower levels of TGF-β than afebrile children.

**Conclusions:**

These results suggest that infection with *Anaplasma* spp. downregulates cytokines including IL-6, IL-22 and TGF-β and that co-infection with *Plasmodium* spp. might have a protective effect on the host, while co-infection with helminths might have a negative effect.

**Graphical Abstract:**

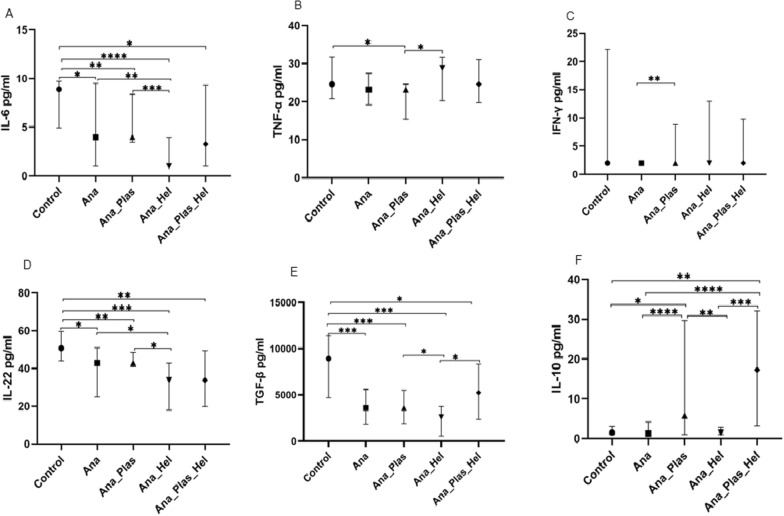

**Supplementary Information:**

The online version contains supplementary material available at 10.1186/s13071-025-06714-1.

## Background

*Anaplasma* like *Plasmodium* is a vector-borne pathogen which can cause human infections. This tick-borne pathogen is a bacterium responsible for many veterinary and human disease worldwide [1, 2]. A significant burden of disease in the Northern Hemisphere has been reported, with around 50,000 human infections caused by these pathogens each year in the USA alone [1]. In Europe, tick-borne diseases are an increasingly serious problem, while in sub-Saharan Africa they are responsible for a significant drop in livestock production and mortality [3–5]. Recently, ticks have been second after mosquitoes as vectors of human pathogens and play an essential role in the transmission of infections such as anaplasmosis [6, 7]. Anaplasmosis is caused by an obligate intracellular bacterium belonging to the Anaplasmataceae family in the order Rickettsiales, whose genera *Ehrlichia* and *Anaplasma* are of interest in human health. The *Anaplasma* genus currently comprises seven formally described species among several others: *Anaplasma marginale, A. centrale, A. ovis, A. platys, A. caudatum, A. bovis* and *A. phagocytophilum* [8]. In addition, *A. phagocytophilum, A. platys, A. bovis* and *Anaplasma capra* have been shown to be pathogenic to humans and have been reported in America, Europe, Asia and Africa [9]. In sub-Saharan Africa, anaplasmosis is generally considered one of the major cattle diseases, while the existence of human infections with Anaplasmataceae has since been demonstrated by serological evidence [10, 11]. This fact highlights the existence of human infections due to Anaplasmataceae in this part of the world. This is supported by the recent results of one of our studies, which reported a high prevalence of asymptomatic *Anaplasma* spp. infections (57.5% by microscopy examination and 86.9% by PCR) among school-aged children in southeastern Gabon [12]. People with asymptomatic Anaplasmataceae infections, due to the absence of signs of clinical illness, are not diagnosed and remain untreated. This situation does not facilitate the implementation of intervention and sanitary control measures. Moreover, like for malaria, these asymptomatic untreated carriers can constitute reservoirs that maintain the chain of transmission. However, infection with *Anaplasma* spp. may present in an uncomplicated clinical form with non-specific symptoms such as fever, chills, malaise, headaches and myalgia as well as non-specific gastrointestinal or respiratory symptoms [13]. In a minority of cases, the disease can progress to life-threatening complications [14, 15].

The immune mechanisms involved in maintaining asymptomatic *Anaplasma* infections and leading to the onset of the first symptoms are not fully understood. Also, asymptomatic infections with *Anaplasma* spp. are still rarely reported, and the basis of the immune response during these has not been described in humans [16, 17]. However, *Anaplasma* spp. is known to affect progenitors of myeloid like erythrocyte cells, monocytic lineages and neutrophils [18]. Recognition of *Anaplasma* by neutrophils and macrophages via the Toll-like receptor (TLR) induces inflammatory responses promoting cytokine release [19]. Among the cytokines released, IFN-γ is thought to prevent an effective antimicrobial response by Th1 and cytotoxic cells [20, 21]. These cytokine-induced mechanisms in response to infection explain the clinical manifestations associated with human granulocytic anaplasmosis. In the acute stage of anaplasmosis (≤ 4 days of illness), patients tend to have the highest IFN-γ, IL-12p70 and IL-2 levels [22]. Also, a pathophysiological role of inflammatory cytokine (IFN-γ, IL-1β, IL-8, IL-6 and TNF-α) response in human anaplasmosis has been reported, particularly in the modulation of hematopoiesis and hematopoietic complications [22]. In addition, suppression of IFN-γ and chemokine responses, including IL-8 and IL-18, in horses infected with *A. phagocytophilum* by dexamethasone has two effects [23]. First, it delays infection-induced limb edema and reduces symptoms. Second, it increases IL-4 and IL-10, suggesting that the Th1 pro-inflammatory response plays a role in aggravating disease severity. Consequently, disease severity may be reduced by modulation of the pro-inflammatory response by anti-inflammatory Th2 cytokines (IL-4 and IL-13) or immunoregulators such as IL-10 and TGF-β.

However, as bacteremia and malaria coinfection is common in sub-Saharan Africa children, it has been shown that co-infection with gram-negative bacteremia and malaria acts to reduce the parasite load [24]. However, there is currently insufficient evidence to determine whether asymptomatic parasitemia is a risk factor for gram-negative bacteremia. In addition, pro-inflammatory cytokines of the Th1 (IFN-γ and TNF-α, IL-6) generally have the Th17 types (IL-17, IL-22 and IL-23) characteristic of malaria infections; however, at parasitemia peak levels as the disease progresses, there is a gradual shift to a Th2 response [25–28], which also is characteristic of helminth infections [29, 30]. At the parasitemia peak, this Th2 response is dominated by upregulation of counter-inflammatory cytokines such as IL-10 and TGF-β [29, 30]. It has also been observed that, in the case of malaria, coinfection with helminths in lethal and resolutive malaria models has contrasting effects [31]. Indeed, the presence of helminths exacerbates mortality and increases the parasitemia peak in normally resolving malaria infections [31]. However, in cases of lethal malaria infections, this tends to move in the opposite direction, with no change in the parasitemia and protective effect against severe malaria [31, 32]. It is therefore likely that the balance between Th1 and Th2 immune responses during polyparasitism is decisive for the clinical course [33–36]. Finally, the inflammatory response of cytokines during asymptomatic infection with *Anaplasma* spp. remains poorly understood and unexplored [22, 37–39]. The aim of this study was therefore to investigate the inflammatory cytokine response during asymptomatic *Anaplasma* spp. infections in schoolchildren and the impact of co-infections with *Plasmodium* spp. and/or helminths.

## Methods

### Study population and sampling

This was a case-control study involving children aged 5 to 17 years (with informed consent) enrolled in five public elementary schools in the city of Franceville, capital of Haut-Ogooué province: Ongouégné, Ondzei, Makana, Application and Ombelé. The study was conducted from March 3 to April 6, 2021. Consent was obtained from parents or legal guardians for all participants. Parameters (such as temperature, weight, age, etc.) were recorded; blood and stool were collected in an EDTA tube and a stool jar, respectively, from each participant. The samples were then sent to the Centre Interdisciplinaire de Recherches Médicales de Franceville (CIRMF). Immediate analyses such as blood count, thick drop and RDT from whole blood were performed. Then, after centrifugation, the plasma was separated from the pellet and stored at − 80 °C; the pellet was kept at − 20 °C for further analysis.

### Various tests and diagnostics performed

#### Hematological analyses

An automated device (Beckman Coulter AcT diff2, Beckman Coulter Corporation, Miami, FL, USA) was used to determine hematological parameters.

#### Diagnosis of *Plasmodium* infection

*Plasmodium* infection was diagnosed using two different methods: rapid diagnostic test (RDT) and microscopy. The RDT was performed using the OptiMAL-IT® test (HRP2) to determine the presence of *Plasmodium* spp., enabling rapid case management. Parasite load was determined by thick drop. Briefly, 10 µl whole blood was smeared on a slide, dried and stained with 10% Giemsa (RAL 555 Kit; RAL Diagnostic, Martillac, France). After washing and drying, the number of *Plasmodium* parasites was determined by two, or at most three, qualified technicians from CIRMF, Gabon [40].

#### Diagnosis of helminthic infection

Diagnosis of helminth infections was carried out according to the slightly modified protocol of Sapero et al. [41]. The stool samples were diluted in 20 ml 0.9% saline. After homogenization, 5 ml of the mixture was taken from various points under the meniscus, placed in a 15-ml Falcon tube and centrifuged at 1500 rpm for 2 min. After discarding the supernatant, the pellet was suspended in 500 µl distilled water. A drop of homogenate was placed on a slide, followed by a drop of merthiolate-lugol solution (1% iodine). The homogenate was then covered between the slide and the coverslip and examined 2 min later at objective × 10 and then × 40 under the microscope. The different intestinal parasites found (designated by helminths in the present study) were *Ascaris lumbricoides*, *Ancylostoma* spp., *Taenia saginata* and *Trichuris trichiura* and protozoans including *Entamoeba coli*, *Endolimax nana*, *Enteromona hominis* and *Giardia* spp. [42].

#### Diagnosis of anaplasmosis infection

This was carried out using the thick drop protocol [40] and nested PCR for genus diagnosis. Slides were examined under a microscope for the presence of bacteria of the Anaplasmataceae family, while PCR amplification enabled detection of *Anaplasma* spp. and *Ehrlichia* spp. For PCR, the first step was to extract DNA. The blood pellet was used. Using the GeneJET genomic purification mini-kit (Thermo Scientific, catalog no. K0782, USA) in accordance with the manufacturer's instructions, DNA was isolated and stored at –20 °C until use. The quality and concentration of extracted DNA were assessed using the NanoDropTM 2000 spectrophotometer (Thermo Fisher Scientific, USA) prior to sample screening. DNA extracts were then amplified using conventional PCR techniques to determine the presence of Anaplasmataceae, including the genera *Anaplasma* and *Ehrlichia* [43, 44]. To this end, primers (TGACAGCGTACCTTTTGCAT for TtAna-F and GTAACAGGTTCG-GTCCTCCA for TtAna-R) targeting the 23S rRNA gene were used to amplify a 191 base pair (bp) amplicon [43, 44]. All Anaplasmataceae-positive samples were amplified by nested PCR using primers specifically targeting the RNA polymerase beta subunit rpoB gene for the *Anaplasma* genus (525 bp) (GCTGTTCCTAGGCTYTCTTACG-CGA for Ana-rpoBF and AATCRAGCCAVGAGCCCCTRTAWGG for Ana-rpoBR) and groEL (GTTGAAAARACTGATGGTATGCA for Ehr-groEL-F and ACACGRTCTTTACG-YTCYTTAAC for Ehr-groEL-R) gene for the *Ehrlichia* genus (590 bp) [45].

Conventional PCR was performed in a Bio-Rad thermal cycler (Applied Biosystems) and from 25 μl of final volume containing 2 μl DNA extracts, 2.5 μl 10X PCR buffer [200 mM Tris–HCl (pH 8.4), 500 mM KCl], 200 μM of each dNTP, 3 mM MgCl2, 20 ρmol of each primer, 2.5 units of Taq DNA polymerase enzyme (Invitrogen, Life Technology) and ultrapure water. Amplification reactions were carried out under the following conditions: an initial denaturation step at 95 °C for 7 min, followed by 35 denaturation cycles of 45 s at 94 °C, 30 s annealing at a corresponding temperature and 1 min extension at 72 °C. A final cycle of extension at 72 °C for 10 min was performed and reactions cooled to 4 °C. Amplification products were visualized on 1.5% agarose gel.

### Inclusion and non-inclusion criteria and study design

All participants whose parents or legal guardians gave informed consent were enrolled. In the present study, participants who were infected with *Anaplasma* spp. alone or co-infected with *Plasmodium* spp. and/or intestinal parasites, as well as uninfected healthy controls, were included. Children infected with *Plasmodium* spp. and intestinal parasites were excluded if they were not infected with *Anaplasma* spp. Among the healthy control children, those with elevated white blood cell levels above normal were also excluded. The participants were then classified into two groups: healthy control children and children infected with *Anaplasma* spp. Then, the *Anaplasma* spp.-infected children were subdivided into three groups (children uninfected with *Anaplasma* spp., those co-infected with *Plasmodium* spp., those co-infected with helminths and those with the tri-infection *Anaplasma* spp., *Plasmodium* spp. and helminths. The *Anaplasma* spp.-infected group was then subdivided into two groups according to afebrile and febrile status.

### Cytokines assay

Circulating levels of ten cytokines including IL-6, IL-10, IL-4, IFN-γ, TNF-α, IL-13, IL-12p70, IL-17A, IL-22 and TGF-β were determined by enzyme-linked immunosorbent assay (ELISA) following the manufacturer's instructions (Bender MedSystems, Vienna, Austria). Optical densities were measured at 450 nm with a reference wavelength of 620 nm in an ELISA plate reader (Stat Fax 3200® Bioblock scientific; Fisher). The detection limit for each cytokine was 2 pg/ml for IL-6, IL-10 and IL-4; 4 pg/ml for IFN-γ, TNF-α, IL-13, IL-12p40 and IL-17; 8 pg/ml for IL-22 and TGF-β. All samples were tested in duplicate, and the data were then entered into Excel to determine the concentrations of the different samples.

### Data processing and analysis

The various results were entered into an Excel database and analyzed statistically. These analyses were carried out using GraphPad Prism 8.0 software. The Agostino & Pearson and Shapiro-Wilk normality tests were used to study the homogeneity of the data distribution. When the data did not follow a normal distribution, the Kruskal-Walis test was used for comparisons between more than two groups. Then, when there was a statistically significant difference or a trend of difference, the Mann-Whitney test was used for pairwise comparisons. The Spearman test was also used to assess the correlation between age, temperature, weight and cytokine levels in infected children. All tests were considered significant for *P* < 0.05.

## Results

### Socio-demographic and para-clinical characteristics

A total of 219 children aged 5 to 17 years were included in the present study, of whom 205 were infected with *Anaplasma* spp. and 14 were uninfected. Infected children had significantly higher white blood cell and lymphocyte levels than in uninfected children [7.0 (6.1–8.4) × 10^3^ cells/mm^3^ vs. 6.0 (5.4–6.8) × 10^3^ cells/mm^3^; *P* = 0.011 and 3.47 (2.76–4.10) × 10^3^ cells/mm^3^ vs. (2.80 (2.31–3.35) × 10^3^ cells/mm^3^, *P* = 0.017, respectively] (Table 1). All other parameters such as age, weight, temperature, red blood cells, hemoglobin, hematocrit, platelets, neutrophils, monocytes, eosinophils and basophils showed similar levels in both groups (*P* > 0.05). Thus, infection with *Anaplasma* spp*.* would modulate certain immune cells such as white blood cells including lymphocytes upwards.

**Table 1:** Demographic and hematological parameters of the included children

Parameters	Children		
	Uninfected (*n* = 14)	Infected (*n* = 205)	*P*
Sex ratio	0.18	1	
Age (years)	9.5 (7–10.25)	10 (8–12)	0.225
Weight (kg)	23.1 (19.8–30.9)	27.3 (22.1–39)	0.055
Temperature (°C)	37.2 (36.9–37.3)	37.1 (36.8–37.4)	0.879
Red blood cells (10^3^ cells/mm^3^); Ref: 3.80–6.00	4.7 (4.3–5.1)	4.6 (4.1–4.8)	0.058
Hemoglobin (g/dl); Ref: 11.5–17.0	12.5 (11.2–13.3)	11.9 (11.1–12.7)	0.317
Hematocrit (%); Ref: 35.0–52.0	34.6 (32.5–37.1)	33.2 (31–35.8)	0.14
Platelets (10^3^/µl); Ref: 150–400	346 (245–378)	283 (214–353)	0.175
White blood cells (10^3^ cells/mm^3^); Ref: 3.50–10.00	6.0 (5.4–6.8)	7.0 (6.1–8.4)	**0.011**
Neutrophils (#); Ref: 1.60–7.00	1.99 (1.44–2.62)	2.32 (1.88–3.18)	0.103
Lymphocytes (#); Ref: 1.00–3.00	2.80 (2.31–3.35)	3.47 (2.76–4.10)	**0.017**
Monocytes (#); Ref: 0.20–0.80	0.55 (0.42–0.68)	0.62 (0.48–0.77)	0.181
Eosinophils (#); Ref: 0.00–0.50	0.19 (0.12–0.48)	0.36 (0.20–0.65)	0.052
Basophils (#); Ref: 0.00–0.15	0.07 (0.04–0.11)	0.08 (0.05–0.11)	0.462

Sex ratio, age, weight, temperature, red blood cell, white blood cell, hemoglobin hematocrit, platelet, neutrophil, lymphocyte, monocyte, eosinophil and basophil parameters in *Anaplasma* spp. infected and uninfected children included in the study. The data in table represent the median (Q2) with the 25th percentile (Q1) to the 75th percentile (Q3). Results were significant when *P* < 0.05. Children whose microscopic and molecular tests were negative for *Anaplasma* spp. were considered uninfected.

### Lower levels of pro- (IL-6 and IL-22) and anti-inflammatory (TGF-β) cytokines in children infected with *Anaplasma* spp.

**Fig. 1:**
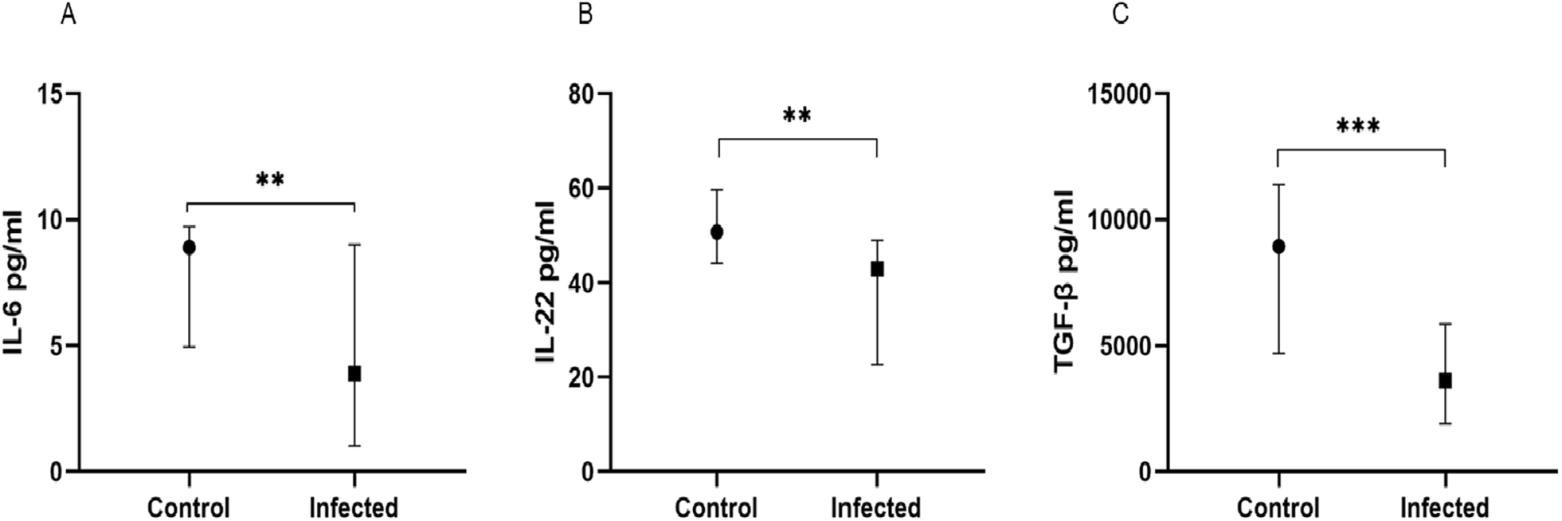
Cytokine levels in infected and uninfected children with *Anaplasma* spp. Plasma concentrations of IL-6 (**A**), IL-22 (**B**) and TGF-β (**C**) were measured in children infected and uninfected with *Anaplasma* spp. Data are represented as median cytokine concentrations in pg/ml with interquartile ranges. The difference between the two groups was determined by the Mann-Whitney test. The difference was significant when *P* < 0.05. **P < 0.01 and ***P < 0.001. *IL* interleukin, *TGF* transforming growth factor

Levels of ten cytokines (IL-6, IL-10, IL-13, IL-4, IL-22, IL-17A, IL-12p70, TNF-α, IFN-γ and TGF-β) were assessed in all children infected and uninfected with *Anaplasma* spp. and compared. Among these cytokines, only IL-6, IL-22 and TGF-β showed statistical differences between the two groups. Indeed, levels were significantly lower in children infected than in those uninfected with *Anaplasma* spp. [3.89 (1.00–9.00) pg/ml vs. 8.90 (4.940–9.73) pg/ml; *P* = 0.002 for IL-6, 42.92 (22.67–48.92) pg/ml vs. 50.67 (44.00–59.63) pg/ml; *P* = 0.003 for IL-22 and 3604 (1899–5860) pg/ml vs. 8937 (4690–11376) pg/ml for TGF-β; *P* = 0.0003] (Fig. 1), respectively. No significant differences were observed between infected and uninfected children for IL-12p70, IL-17A, IFN-γ, TNF-α, IL-4, IL-10 and IL-13 (*P* > 0.05, data not shown). All this suggests that *Anaplasma* infection induces a decrease in the production of certain pro- (IL-6 and IL-22) and anti-inflammatory (TGF-β) cytokines.

### Effect of *Plasmodium* spp. and helminths on cytokines produced during *Anaplasma* spp. infection

To assess the impact of parasite co-infections on cytokine production during *Anaplasma* spp. infection, the group of infected children was subdivided into four groups: children with *Anaplasma* spp. mono-infection (*n* = 93), children with *Anaplasma* spp. and *Plasmodium* spp. co-infection (*n* = 65), children with *Anaplasma* spp. and helminth co-infection (*n* = 18) and children with *Anaplasma* spp., *Plasmodium* spp. and helminth co-infection (*n* = 29) and compared with healthy control children (*n* = 14).

Taken together, comparison between cytokine levels in these groups showed that there was a significant difference between groups for TGF-β, IL-6, IL-10 and IL-22 (*P* = 0.001, *P* = 0.0003, *P* < 0.0001 and *P* = 0.003, respectively) and that there was a trend of difference for TNF-α and IFN-γ (*P* = 0.059 and *P* = 0.082, respectively). However, IL-4, IL-12p70, IL-13 and IL-17A showed no difference (*P* > 0.05).

**Fig. 2:**
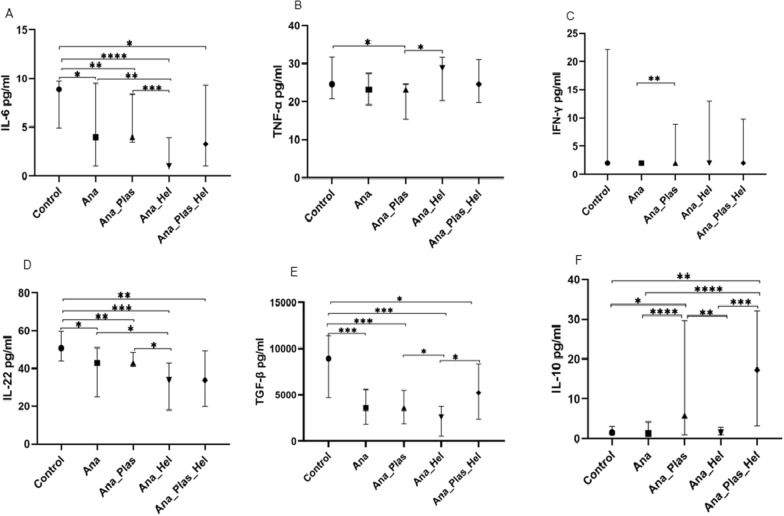
Effect of coinfection with *Plasmodium* spp. and helminths on cytokines levels during *Anaplasma* spp. infection. Levels of IL-6 (**A**), TNF-α (**B**), IFN-γ (**C**), IL-22 (**D**), TGF-β (**E**) and IL-10 (**F**). Ana: *Anaplasma* spp., Ana_Plas: co-infection with *Anaplasma* and *Plasmodium,* Ana_Hel: co-infection with *Anaplasma* and helminths, Ana_Plas_Hel: co-infection with *Anaplasma, Plasmodium* and helminths. Children were either uninfected (healthy controls), mono-infected with *Anaplasma* spp. or co-infected with *Plasmodium* spp. and/or helminths. Cytokine concentrations were measured in children's plasma using the ELISA technique. Data are represented as medians of cytokine concentrations in pg/ml with interquartile ranges. The Kruskal-Wallis test was used for comparisons of more than two groups, and when the difference was significant, the Mann-Whitney test was used for pairwise comparisons. *P < 0.05, **P < 0.01, ***P < 0.001 ****P < 0.0001. *IL* interleukin, *TNF* tumor necrosis factor, *IFN* interferon, *TGF* transforming growth factor

The two-by-two group comparison showed, as expected, that children mono-infected with *Anaplasma* spp. had significantly more decreased levels of IL-6, IL-22 and TGF-β than healthy controls [3.97 (1.00–9.49) pg/ml vs. 8.90 (4.94–9.73) pg/ml; *P* = 0.016 for IL-6, 42.92 (24.89–50.95) pg/ml vs. 50.67(44.00–59.63) pg/m; *P* = 0.013 for IL-22 and 3604 (1839–5591) pg/ml vs. 8937 (4690–11376) pg/ml; *P* = 0.0005 for TGF-β, respectively] (Fig. 2A, D, E). These mono-infected children also had significantly lower levels of IL-10 and IFN-γ than children co-infected with *Anaplasma* spp. and *Plasmodium* spp. [1.26 (1.00–4.18) pg/ml vs. 5.80 (1.00–29.68) pg/ml; *P* < 0.0001, and 2.00 (2.00–2.00) pg/ml vs. 2.00 (2.00–8.85) pg/ml; *P* = 0.004, respectively] (Fig. 2C, F). Furthermore, these mono-infected children had significantly higher levels of IL-6 and IL-22 than children co-infected with *Anaplasma* spp. and helminths [3.97 (1.00–9.49) pg/ml vs. 1.00 (1.00–3.97) pg/ml; *P* = 0.003 for IL-6 and 42.92 (24.89–50.95) pg/ml vs. 33.78 (17.94–42.92) pg/ml; *P* = 0.020 for IL-22; respectively] (Fig. 2A, D). Also, these children mono-infected with *Anaplasma* spp. showed significantly more decreased IL-10 levels than those co-infected with *Anaplasma* spp., *Plasmodium* spp. and helminths [1.00 (1.26–4.18) vs. 17.28 (3.21–32.12) pg/ml; *P* < 0.0001] (Fig. 2F).

On the other hand, the results showed that healthy controls also had significantly higher levels of IL-6, IL-22 and TGF-β than children co-infected with *Anaplasma* spp. and *Plasmodium* spp. (*P* = 0.006, *P* = 0.009 and *P* = 0.0004, respectively), children co-infected with *Anaplasma* spp. and helminths (*P* < 0.0001, *P* = 0.0002 and *P* = 0.0003, respectively) and children co-infected with *Anaplasma* spp., *Plasmodium* spp. and helminths (*P* = 0.013, *P* = 0.005 and *P* = 0.040, respectively) (Fig. 2A, D, E). These control children had significantly higher levels of IL-10 and TNF-α than children co-infected with *Anaplasma* spp. and *Plasmodium* spp. (*P* = 0.019 and *P* = 0.030, respectively) and also significantly higher levels of IL-10 than children co-infected with *Anaplasma* spp., *Plasmodium* spp. and helminths (*P* = 0.001) (Fig. 2B, F).

Children co-infected with *Anaplasma* spp. and *Plasmodium* spp. had significantly higher levels of IL-6, IL-22, TGF-β and IL-10 than those co-infected with *Anaplasma* spp. and helminths (*P* = 0.0004, *P* = 0.025, *P* = 0.045 and *P* = 0.006, respectively) (Fig. 2A, D, E, F); however, surprisingly, enough TNF-α levels were significantly higher (*P* = 0.021) (Fig. 2B).

Finally, as expected, children co-infected with *Anaplasma* spp., *Plasmodium* spp. and helminths showed significantly higher TGF-β and IL-10 levels than those co-infected only with *Anaplasma* spp. and helminths (*P* = 0.031; *P* = 0.0004) (Fig. 2E, F).

### Elevated levels of IFN-γ and decreased levels of TNF-α and TGF-β in febrile compared to afebrile children all infected with *Anaplasma* spp.

**Fig. 3:**
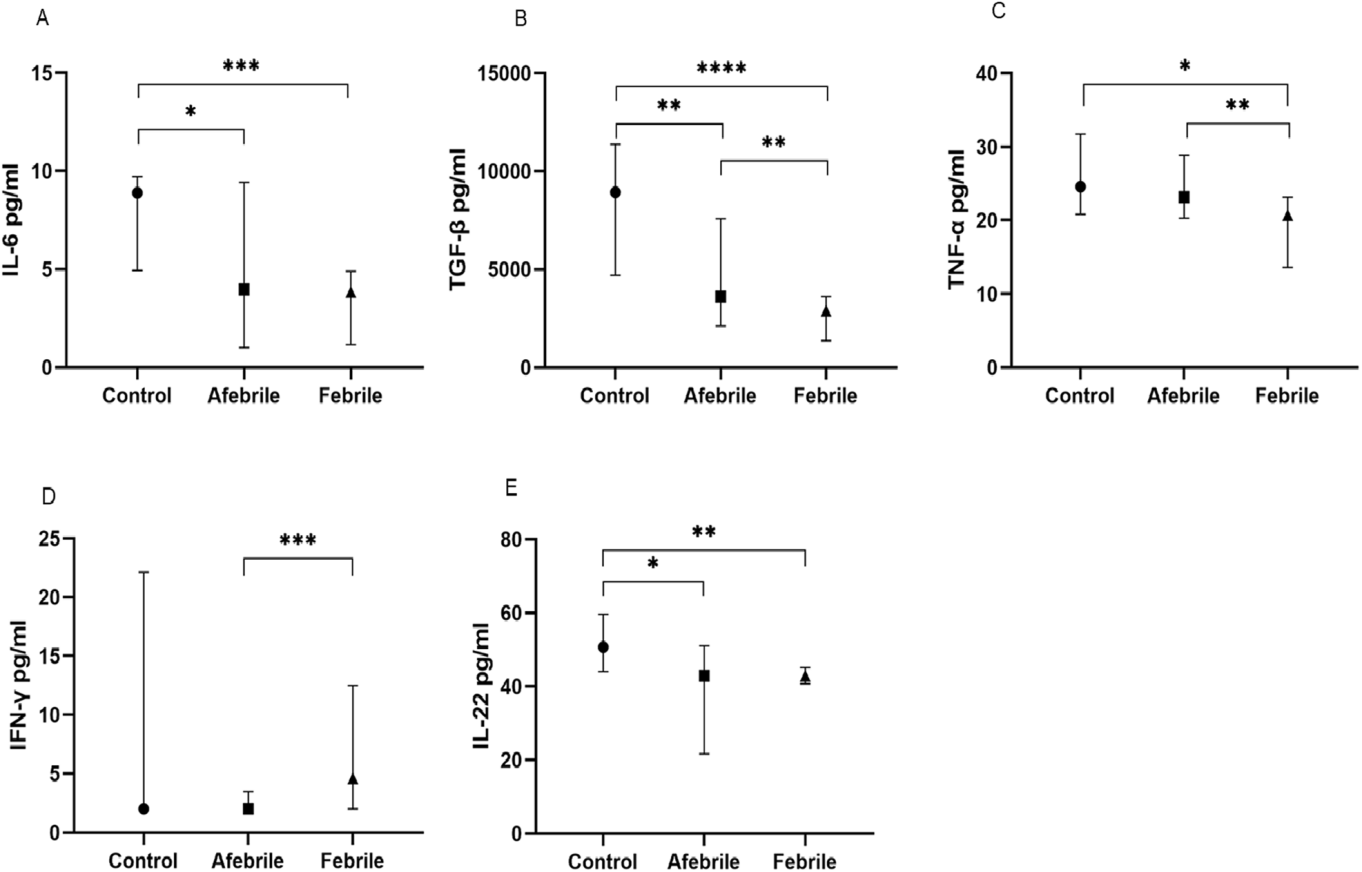
Cytokine levels released according to febrile and afebrile state of children. Levels of IL-6 (**A**), TGF-β (**B**) TNF-α (**C**), IFN-γ (**D**) and IL-22 (**E**) in afebrile and febrile children infected with *Anaplasma* spp. and uninfected control children were compared. Cytokine concentrations were measured from children's plasma using the ELISA technique. Data are represented as medians of cytokine concentrations in pg/ml with interquartile ranges. The Kruskal-Wallis test was used for comparisons of more than two groups, and when the difference was significant, the Mann-Whitney test was used for pairwise comparisons. *P < 0.05, **P < 0.01, ***P < 0.001, ****P < 0.0001. *IL* interleukin, *TNF* tumor necrosis factor, *IFN* interferon, *TGF* transforming growth factor

To assess the effect of fever on cytokine production in our *Anaplasma* spp.-infected children, this group of children was subdivided into two groups, afebrile (*n* = 175) and febrile children (*n* = 44), and compared with healthy control children (*n* = 14). Analysis showed that febrile children had significantly higher levels of IFN-γ and more decreased levels of TGF-β and TNF-α than afebrile children [4.62 (2.000–12.50) pg/ml vs. 2.000 (2.00–3.46) pg/ml; *P* = 0.0003 for IFN-γ and 2899 (1375–3604) pg/ml vs. 3604 (2117–7581) pg/ml; *P* = 0.002 for TGF-β and (20.71 (13.57–23.14) pg/ml vs. 23.14 (20.29–28.86) pg/ml; *P* = 0.002), respectively] (Fig. 3B, C, D).

Unsurprisingly, afebrile children had significantly more decreased levels of IL-6, IL-22 and TGF-β than healthy controls [3.97 (1.000–9.43) pg/ml vs. 8.90 (4.94–9.73) pg/ml; *P* = 0.013 for IL-6, 42.92 (21.61–51.06) pg/ml vs. 50.67 (44.00–59.63) pg/ml; *P* = 0.011 for IL-22 and 3604 (2117–7581) pg/ml vs. 8937 (4690–11376) pg/ml; *P* = 0.004 for TGF-β, respectively] (Fig. 3A, B, E).

However, surprisingly the febrile children also had significantly more decreased levels of IL-6, TGF-β, TNF-α and IL-22 than the control children [3.84 (1.16–4.89) pg/ml vs. 8.90 (4.94–9.73) pg/ml; *P* = 0.0002 for IL-6, 2899 (1375–36.4) pg/ml vs. 8937(4690–11376) pg/ml; *P* < 0.0001 for TGF-β, 20.71 (13.57–23.14) pg/ml vs. 24.59 (20.83–31.76) pg/ml; *P* = 0.011 for TNF-α and 42.92 (40.73–45.11) pg/ml vs. 50.67 (44.00–59.63) pg/ml; *P* = 0.002 for IL-22, respectively] (Fig. 3A, B, C, E). The other cytokines measured showed no significant difference (data not shown).

### Cytokine correlations in children infected with *Anaplasma* spp.

Data revealed that age was positively correlated with weight and IFN-γ (*r* = 0.82; *P* < 0.00001 and *r* = 0.23; *P* = 0.001, respectively) and was negatively correlated with IL-6, IL-22 and TGF-β (*r* = − 0.36; *P* < 0.0001, *r* = − 0.24; *P* < 0.001, *r* = − 0.22; *P* = 0.001, respectively) (Table 2). In addition, weight was negatively correlated with IL-6 and TGF-β (*r* = − 0.26; *P* < 0.0001, *r* = − 0.20; *P* = 0.003, respectively) and temperature was positively with IFN-γ and negatively correlated with TGF-β (*r* = 0.24; *P* = 0.0002, *r* = − 0.23; *P* = 0.001). IL-22 was strongly correlated with IL-6 (*r* = 0.76; *P* < 0.00001), which in turn was weakly correlated with IL-10 (*r* = 0.32; *P* < 0.00001). Furthermore IL-17A was weakly correlated with IL-4, IL-10 and IL-12p70 (*r* = 0.30; *P* < 0.00001, *r* = 0.22; *P* = 0.001 and *r* = 0.30; *P* < 0.00001, respectively) while TGF-β was correlated with IL-6 and IL-22 (*r* = 0.44; *P* < 0.00001 and *r* = 0.32; *P* < 0.00001, respectively).

**Table 2:** Correlation between age, weight, temperature and cytokines

Parameters	Age	Weight	Temperature	IL-4	IL-6	IL-10	IL-12p70	IL-13	IL-17A	IL-22	IFN-γ	TNF-α	TGF-β
Age	1												
Weight	**0.82**	1											
Temperature	0.12	0.15	1										
IL-4	− 0.0006	0.02	− 0.01	1									
IL-6	− **0.36**	− **0.26**	− 0.06	0.03	1								
IL-10	− 0.13	− 0.19	0.02	0.11	**0.32**	1							
IL-12p70	0.03	0.018	0.02	− 0.005	− 0.08	0.07	1						
IL-13	− 0.001	− 0.003	0.02	− 0.005	− 0.04	− 0.08	− 0.005	1					
IL-17A	− 0.02	− 0.003	0.06	**0.30**	0.03	**0.22**	**0.30**	− 0.01	1				
IL-22	− **0.24**	− 0.15	− 0.06	0.12	**0.76**	0.18	− 0.07	− 0.04	0.12	1			
IFN-γ	**0.23**	0.18	**0.24**	0.14	− 0.09	0.09	− 0.05	− 0.05	0.06	0.04	1		
TNF-α	− 0.12	− 0.16	− 0.14	− 0.04	− 0.17	0.04	0.03	− 0.12	0.09	− 0.19	− 0.005	1	
TGF-β	− **0.22**	− **0.2**	− **0.23**	0.03	**0.44**	0.14	0.02	0.09	0.003	**0.32**	-0.13	− 0.11	1

Spearman's correlation analysis was performed on age, temperature, weight and cytokines in children infected with *Anaplasma* spp. The correlation coefficient is given and is strong for rho = 0.7 to 1, moderate for rho = 0.5 to 0.7 and weak for rho = 0.2 to 0.5, and for a value close to 0, there is no correlation. The statistically significant correlations with *P*-value < 0.05 are presented in bold

## Discussion

This study examined inflammatory cytokine levels in children infected and uninfected with *Anaplasma* spp. Infected children showed no clinical signs associated with human anaplasmosis (except for an above-normal temperature in a few children); however, they tended to have significantly lower red blood cell levels than uninfected children, although still at normal values. This is because various pathogens of the *Anaplasma* genus are known to infect humans, such as *Anaplasma capra* and *Anaplasma ovis*, or any other unknown species targeting erythrocyte cells or red blood cells and induce destruction of red blood cells [46]. On one hand, infected erythrocytes could be destroyed by the cell effectors of the immune system and by the lysis of infected erythrocytes releasing the multiplied bacteria into the bloodstream. Another possible explanation could be an infection by another *Anaplasma* species that does not target red blood cells, such as *A. phagocytophilum*. Indeed, in an experimental murine model, infection by *A. phagocytophilum* was shown to induce anemia early in the course of infection (up to 7 days post-infection) by reducing the number of red blood cells [47]. In addition, children infected with *Anaplasma* spp. showed a significant increase in some hematological parameters, including white blood cells and lymphocytes. This could suggest a mechanism put in place by the host organism to fight the infection. All of this is consistent with the literature, as infection with *Anaplasma* spp*.* has been reported to affect hematological parameters, with inflammation-mediated changes in hematopoietic subsets resulting in mild anemia [47].

Infected children also had significantly lower levels of pro- (IL-6 and IL-22) and anti-inflammatory (TGF-β) cytokines than uninfected children, suggesting that the pathogen may be responsible for the downregulation of these cytokines. Indeed, it has been shown that Anaplasmataceae of the genus *Anaplasma* do not express peptidoglycans or lipopolysaccharides (LPS) [47, 48]. As the latter molecules are generally identified by the innate immune system via recognition receptors expressed by macrophages or neutrophils to eliminate pathogens, their absence would have the direct consequence of delaying the activation of the innate response against these pathogens. This would result in bacteria persisting in host cells without triggering an effective innate immune response. Moreover, as the *Anaplasma* spp. genus has a tropism for different cells of this same innate immunity, its presence within them could induce an alteration in the response of these cells, reflected in the lower cytokine levels observed in our infected children. In addition, other studies have shown that tick saliva possesses immunomodulatory properties that inhibit inflammation at the vector-host interface and attenuates *A. phagocytophilum*-induced cytokine secretion by macrophages in a murine model [49]. Furthermore, a strong positive correlation between IL-6 and IL-22 and their weak associations with TGF-β suggest that these cytokines are produced in a competitive manner. These results support the hypothesis that one of the survival strategies of *A. phagocytophilum* is to downregulate the host cell's pro-inflammatory response [50]. However, another hypothesis raised could be that *Anaplasma* spp. pathogen is responsible for the downregulation of the anti-inflammatory cytokine TGF-β. Furthermore, these results showed that cytokines such as IFN-γ, TNF-α, IL-12p70, IL-17A, IL-10, IL-4 and IL-13 showed no difference between infected and uninfected groups, suggesting that the expression of these cytokines is not induced or is induced later in time.

Comparison of mono- and co-infected children revealed that the group of children co-infected with *Anaplasma* spp. and *Plasmodium* spp. had significantly higher concentrations of the cytokines IL-10 and IFN-γ than the group mono-infected with *Anaplasma* spp., suggesting a protective effect of *Plamodium* spp. Indeed, an increased level of Th1 cytokines such as IFN-γ and Th2 (IL-10) will induce a pro-inflammatory response participating in pathogen clearance by effector cells of cellular and humoral adaptive immunity mediated by T and B cells, respectively [51, 52]. However, children co-infected with *Anaplasma* spp. and helminths also showed significantly lower levels of IL-6 and IL-22 than those mono-infected with *Anaplasma* spp., suggesting a negative effect of the presence of helminths. Also, the significantly lower IL-10 levels observed in the group of children mono-infected with *Anaplasma* spp. compared to those co-infected with *Anaplasma* spp., *Plasmodium* spp. and helminths are in line with the protective effects of *Plasmodium* spp. during the co-infection.

The effect of fever on cytokine production in children infected with *Anaplasma* spp. was also assessed. The results showed that asymptomatic infections with *Anaplasma* spp. induce a decrease in IL-6, IL-22 and TGF-β secretion; however, in febrile cases there is a reversal of the cytokine response resulting in significantly elevated levels of IFN-γ. Our results are similar to those observed in other studies on *A. phagocytophilum* infection, where it has been reported that these infections stimulate a Th1/pro-inflammatory cytokine response in humans and that IFN-γ was particularly elevated in patients with human anaplasmosis [53]. This cytokine can be secreted by cells such as NK and NKT but also by CD8 + T cells, a component of adaptive immunity [54]. However, it is a key cytokine in the early acute phase of the disease, activating macrophage production of nitric oxide, reactive oxygen species and TNF-α, as well as phagocytosis, leading to cytotoxic effects [47]. Also, it has been stipulated that in vivo responses to *Anaplasma* are dominated by IFN-γ [54], and during infection by bacteria, exogenous pyrogens induced host cells, primarily macrophages, to produce and release endogenous pyrogens such as IFN-γ and interleukin-1 [55, 56]. The signal transmitted by endogenous pyrogens to the hypothalamic thermoregulatory center induces the synthesis of prostaglandin 2, which then acts on the hypothalamus to raise body temperature reflected in fever [57].

As with any work, there are a number of limitations. This study was mainly limited by the number of healthy controls, which was too small compared to the cases studied. In addition, cytokine responses in children mono-infected with *Plasmodium* spp. and mono-infected with helminths were not considered in the present study. Nevertheless, the results of this work show that, in general, early infections with *Anaplasma* spp. induce a decrease in cytokines. However, as temperature rises, there is a reversal of the cytokine response, reflected here by significantly elevated IFN-γ levels.

## Conclusions

This study demonstrated that in healthy school children, asymptomatic infection with *Anaplasma* spp. results in mild hematological variations and reduced production of pro- and anti-inflammatory cytokines (IL-6, IL-22 and TGF-β). Moreover, during co-infection with *Anaplasma* spp. and *Plasmodium* spp., the latter proved to be a host-protective factor in contrast to helminths. These intestinal parasites were found to have a negative effect on cytokine production. However, it would be interesting to establish an inflammatory profile by *Anaplasma* species to better discriminate between the species infecting humans and also to determine whether the variations in pro- and anti-inflammatory cytokine production observed here have an impact on the homeostasis of the inflammatory response.

## Supplementary Information

Supplementary Material 1.

## Data Availability

The database associated with this study is directly accessible through contact with the corresponding author.
