# Supplementary material for: Cytokine pattern during asymptomatic Anaplasma spp. infections and effect of co-infections by malaria and helminths in schoolchildren of Franceville, southeastern Gabon
Source: Parasit Vectors. 2025 Mar 27;18:118. doi: 10.1186/s13071-025-06714-1 (PMC11948865; doi:10.1186/s13071-025-06714-1)
Supplement: Supplementary file 1 — Supplementary Material 1. [file 13071_2025_6714_MOESM1_ESM.docx]

| Parameters |  | Children | | |
| --- | --- | --- | --- | --- |
|  | Control (14) | Afebrile (n = 175) | Febrile (n = 44) | *P* |
| Sex ratio | 0.18 | 0.99 | 0.69 |  |
| Age (years) | 9.5 (7-10.25) | 10 (8-12) | 11 (8-13) | 0.077 |
| Weight (Kg) | 23.1 (19.8-30.9) | 26.8 (22.1-36.3) | 35 (21.4-45.8) | **0.030** |
| Temperature (°C) | 37.2 (36.9-37.3) | 37.0 (36.7-37.2) | 37.6 (37.5-37.7) | **<0.0001** |
| Red blood cells (10^3^ cells/mm^3^); Ref: 3.80-6.00 | 4.7 (4.3-5.1) | 4.4 (4.1-4.8) | 4.6 (4.2-4.8) | 0.124 |
| Hemoglobin (g/dL); Ref: 11.5-17.0 | 12.5 (11.2-13.3) | 11.8 (11.1-12.7) | 12.3(11.2-13.3) | 0.242 |
| Hematocrit (%); Ref: 35.0-52.0 | 34.6 (32.5-37.1) | 33.1 (31.0-36.0) | 33.8 (31.0-36.4) | 0.295 |
| Platelets (10^3^/µL); Ref: 150-400 | 346 (245-378) | 290 (212-360) | 292 (225-340) | 0.439 |
| White blood cells (10^3^ cells/mm^3^); Ref: 3.50-10.00 | 6.0 (5.4-6.8) | 7.0 (5.9-8.1) | 7.9 (6.5-9.4) | **0.001** |
| Neutrophils (#); Ref: 1.60-7.00 | 1.99 (1.44-2.62) | 2.20 (1.77-3.04) | 2.91 (2.05-3.42) | **0.005** |
| Lymphocytes (#); Ref: 1.00-3.00 | 2.80 (2.31-3.35) | 3.34 (2.68-4.01) | 3.56 (2.78-4.23) | **0.039** |
| Monocytes (#); Ref: 0.20-0.80 | 0.55 (0.42-0.68) | 060 (0.47-0.76) | 0.65 (0.49-0.81) | 0.334 |
| Eosinophils (#); Ref: 0.00-0.50 | 0.19 (0.12-0.48) | 0.34 (0.18-0.63) | 0.45 (0.24-0.82) | 0.104 |
| Basophils (#); Ref: 0.00-0.15 | 0.07 (0.04-0.11) | 0.08 (0.06-011) | 0.07 (0.05-0.11) | 0.728 |
